# Advances in oncolytic herpes simplex virus and adenovirus therapy for recurrent glioma

**DOI:** 10.3389/fimmu.2023.1285113

**Published:** 2023-11-02

**Authors:** Mingming Hu, XuLiang Liao, Yi Tao, Yaohui Chen

**Affiliations:** ^1^ Institute of Thoracic Oncology, West China Hospital, Sichuan University, Chengdu, China; ^2^ Frontiers Science Center for Disease-Related Molecular Network, West China Hospital, Sichuan University, Chengdu, China; ^3^ Department of Thoracic Surgery, West China Hospital, Sichuan University, Chengdu, China

**Keywords:** oncolytic viruses, cancer therapy, recurrent gliomas, oncolytic herpes simplex virus, adenovirus therapy

## Abstract

Recurrent glioma treatment is challenging due to molecular heterogeneity and treatment resistance commonly observed in these tumors. Researchers are actively pursuing new therapeutic strategies. Oncolytic viruses have emerged as a promising option. Oncolytic viruses selectively replicate within tumor cells, destroying them and stimulating the immune system for an enhanced anticancer response. Among Oncolytic viruses investigated for recurrent gliomas, oncolytic herpes simplex virus and oncolytic adenovirus show notable potential. Genetic modifications play a crucial role in optimizing their therapeutic efficacy. Different generations of replicative conditioned oncolytic human adenovirus and oncolytic HSV have been developed, incorporating specific modifications to enhance tumor selectivity, replication efficiency, and immune activation. This review article summarizes these genetic modifications, offering insights into the underlying mechanisms of Oncolytic viruses’ therapy. It also aims to identify strategies for further enhancing the therapeutic benefits of Oncolytic viruses. However, it is important to acknowledge that additional research and clinical trials are necessary to establish the safety, efficacy, and optimal utilization of Oncolytic viruses in treating recurrent glioblastoma.

## 1 Introduction

Gliomas, specifically glioblastomas (GBM), represent a majority of central nervous system malignancies and are the most common primary brain tumors. Gliomas originate from glial or progenitor cells and are classified into four grades by the World Health Organization (WHO). Glioblastoma (GBM), the most aggressive primary brain tumor in adults, accounts for 16% of all central nervous system tumors (1). It is classified as a grade 4 glioma according to the WHO grading system (2). The standard treatment for GBM includes surgical therapy, radiation therapy, and temozolomide (TMZ) therapy (3). Combining TMZ with radiation therapy can raise the 2-year survival rate to 26.5%, compared to a lower rate of 10.4% with radiation therapy alone (4). However, despite the use of surgical intervention, postoperative radiotherapy, and chemotherapy, GBM remains highly invasive, leading to metastasis, recurrence, and mortality (5). The median survival time for GBM patients is approximately 15 months (1), with minimal likelihood of resurgery for relapsed cases (6–9). Recurrent glioblastoma (rGBM) presents a significant challenge in neurooncology, characterized by increased tumor cell density, neovascularization, blood-brain barrier disruption, permeability, tortuous neovascularization, uneven thickness, and slow blood flow. The pathophysiological mechanism underlying pseudoprogression remains unclear; however, it is believed to involve vascular endothelial, blood-brain barrier, and oligodendrocyte damage, leading to local inflammation, increased blood-brain barrier permeability, and vasogenic edema, resulting in abnormal lesion enhancement on imaging. Surgical resection is often impractical for recurrent tumors, which demonstrate reduced therapy responsiveness and invasion into functional brain areas (4–10). Consequently, median overall survival (mOS) after relapse is approximately 6 months, with no established standard treatment for rGBM, leading to patient mortality within 12-15 months of initial diagnosis (10, 11). RGBM often exhibits resistance to temozolomide (TMZ, a DNA alkylating agent), the standard GBM chemotherapy agent (4). Despite advancements in genetic studies of GBM, no molecular targeting agent has been identified to prolong OS in patients with rGBM.

Tumor immunotherapy is a promising approach to activating specific immune responses against cancer cells within the body, offering the advantages of targeted, efficient treatment with reduced harm to healthy tissues. Unlike conventional methods like surgery, targeting, radiotherapy, and chemotherapy, immunotherapy does not directly eliminate cancer cells. Instead, it mobilizes immune cells capable of recognizing tumors, enhances the body’s immune system’s ability to combat cancer, and relies on these cells to indirectly control and eliminate cancer cells. This strategy has minimal side effects, ensuring safety and efficacy (12). Immunotherapy, including immune checkpoint inhibitors, has shown efficacy in clinical trials for various tumor types (13). However, its effectiveness in patients with recurrent glioma is limited due to factors such as the tumor heterogeneity of GBM, the presence of the blood-brain barrier, and the immunosuppressive nature of the tumor microenvironment (TME)^[^ (14). Glioma, a tumor with heterogeneous characteristics including proliferative potential, invasiveness, histological grade, and clinical behavior, presents a significant challenge for immunotherapy (15). Studies have highlighted the obstacles caused by glioma cells’ immune evasion mechanisms, such as antigen loss or downregulation, which hinder vaccine therapy and CAR-T cell therapy (16). Moreover, the glioma tumor microenvironment contains more immunosuppressive cells than immune effector cells, contributing to the establishment of an immunosuppressive state that promotes glioma growth, invasion, and metastasis (17). Conversely, oncolytic virus (OVs) therapy exhibits promise in early clinical trials for GBM. OVs can selectively infect and destroy tumor cells while modulating the immune system to enhance the anti-tumor response (18).

OVs represent a form of immunotherapy that selectively infect and destroy cancer cells, leading to the release of infectious virus particles that contribute to the destruction of residual tumors. These viruses can impede cancer cell replication or be genetically modified to specifically target and eliminate them. Moreover, OVs have the ability to activate the suppressed immune system, resulting in an adaptive anti-tumor immune response while suppressing tumor growth (19). Both preclinical and clinical trials have evaluated OVs derived from herpes simplex virus-1 (HSV-1), adenovirus (Ad), Newcastle disease virus (NDV), and reovirus (RV) for the treatment of rGBM, demonstrating promising therapeutic effects. However, further clinical trials are necessary to validate these findings.

**Figure 1:**
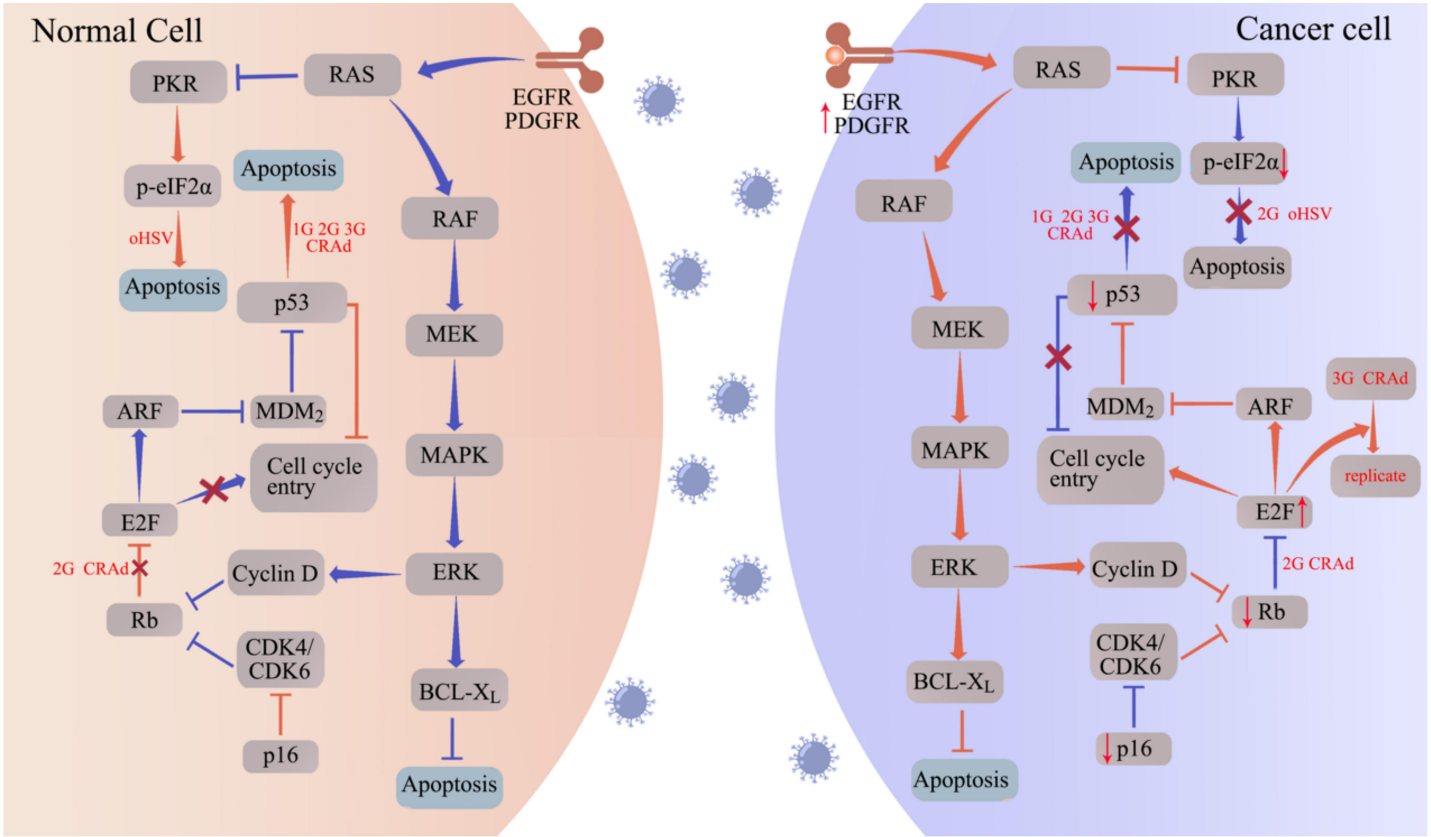
oHSV and CRAd replicate specifically in tumor cells by targeting tumor-associated pathways. Aberrantly expressed proteins in tumor cells promote the replication and oncolytic activity of oHSV and CRAd. In normal cells (left panel), the cell cycle is regulated by proteins such as protein kinase R (PKR), p16, retinoblastoma (Rb), and the tumor suppressor p53. Upon infection with oHSV and CRAd, these cell cycle regulators facilitate apoptosis, hindering viral replication. Conversely, cancer cells often exhibit disruptions in these cell cycle regulators, such as p53 and Rb mutations, to support uncontrolled proliferation. Consequently, when infected with oHSV and CRAd, cancer cells fail to initiate the apoptotic program, allowing for viral replication within the tumor cells. The abbreviations used in the figure are as follows: 1G/2G/3G oHSV refers to first generation, second generation, and third-generation oHSV respectively; 1G/2G/3G CRAd stands for first generation, second generation, and third-generation CRAD; CDK represents cyclin-dependent kinase; EGFR denotes epidermal growth factor receptor; ERK signifies extracellular signal-regulated kinase; MAPK refers to mitogen-activated protein kinase; MEK stands for MAPK/ERK kinase; PDGFR represents platelet-derived growth factor receptor.

This review aims to provide an overview of the oncolytic mechanisms of different OVs, including oHSV, CRAd, and others (**Figure 1**). Additionally, we discuss the survival benefits and safety profiles based on major preclinical and clinical trials of oncolytic viruses in glioma, specifically rGBM (**Table 1**). Currently, PVS-RIPO and DNX-2401 have received fast track designation from the US Food and Drug Administration (FDA), while G47Δ has been approved by the Japanese Ministry of Health, Labor and Welfare for the treatment of malignant glioma. Furthermore, numerous other OVs have demonstrated significant anti-tumor potential in both preclinical and clinical trials. Our goal is to provide a reference for researchers involved in the development of novel OVs, facilitate improved clinical trials for OVs, and offer valuable recommendations for the application of OVs in glioma treatment.

**Table 1:** OVs clinical trials.

Trial Number	Phase	Virus	Other Treatments	Target	Status	Start Date	Country	Report	numbers
	I	G207		Brain malignant glial tumor	completed	Feb, 1998 - May 1999			21
**NCT00157703**	Ib	G207		recurrent/progressive malignant glioma.	completed	Jan, 2002 - Aug, 2003	USA	Mol Ther.2014 May	6
**NCT02457845**	I	G207	radiation (5 Gy)	Recurrent Supratentorial Brain Tumors in Children	Active, not recruiting	May 2016-	USA		13
**NCT02031965**	I	HSV1716	dexamethasone; therapeutic conventional surgery	Refractory or Recurrent HGG	Terminated	Dec 2013-May 2016	USA		2
**NCT03152318**	I	rQNestin34.5v.2	Cyclophosphamide(CPA)	recurrent malignant glioma	Recruiting	July 18, 2017-	USA		62
**UMIN-CTR: UMIN000002661**	I/II	G47Δ		recurrent or progressive glioblastoma	completed	Nov, 2009- Nov, 2014	Japan	2022 Jul 21.	13
**UMIN-CTR: UMIN000015995**	II	G47Δ		Residual or recurrent glioblastoma	Active, not recruiting	Dec, 2014-	Japan		
	I	ONYX-015		malignant glioma	completed	Jan, 2000 - May 2002	USA	2004年11月	24
	II	ONYX-015	5-FU; cisplatin	recurrent head and neck cancer	completed		USA	2000年8月	37
**NCT00805376**	I	DNX-2401		Recurrent Malignant GBM	completed	Feb, 2009-Feb, 2015	USA	2018.5	37
**NCT03178032**	I/II	DNX-2401		Diffuse pontine GBM (DIPG)	completed	May, 2017-Jan, 2021	Spain		12
**NCT01956734**	Ib	DNX-2401	TMZ	Glioblastoma at First Recurrent	completed	Sep, 2013-Mar, 2017	Spain		31
**NCT02197169**	Ib	DNX-2401	IFN-γ	GBM and gliosarcoma (GS)	completed	July, 2014-July 2018	USA		37
**NCT03714334**	I	DNX-2440		Patients with first or second recurrence of GBM	Terminated (Break of stock)	Oct, 2018–	Spain		16
**NCT02798406**	II	DNX-2401	pembrolizumab	GBM and GS	completed	Jun 14, 2016- Jul 15, 2021	USA and Canada	2023 Jun	49
**NCT02444546**	I	Pelareore	GM-CSF	Pediatric Patients with Relapsed or Refractory Brain Tumors	Completed	Jun 21, 2015-Nov, 2022	USA		6
**NCT01491893**	I	Pelareore		Recurrent WHO Grade IV Malignant Glioma	Completed	Apr, 2012-Oct, 2021	USA	2018.7	61
**NCT03043391**	I	PVSRIPO		recurrent WHO grade III or IV malignant glioma in pediatric patients	Active, not recruiting	Dec, 2017-	USA		12
**NCT02986178**	II	PVSRIPO		recurrent WHO grade IV malignant glioma in adults	Active, not recruiting	June 1, 2017-	USA		122
**NCT01470794**	I	Toca 511	Toca FC	rHGG	Completed	February 2012-April 12, 2016	USA	2018 Sep	58
**NCT01156584**	I	Toca 511	Toca FC	rHGG	Completed	July 2010-August 18, 2016	USA		54
**NCT01985256**	I	Toca 511	Toca FC	recurrent or progressive Grade III or Grade IV GBM	Completed	February 2014-March 3, 2016	USA		17

## 2 Oncolytic herpes simplex virus (oHSV)

**Figure 2:**
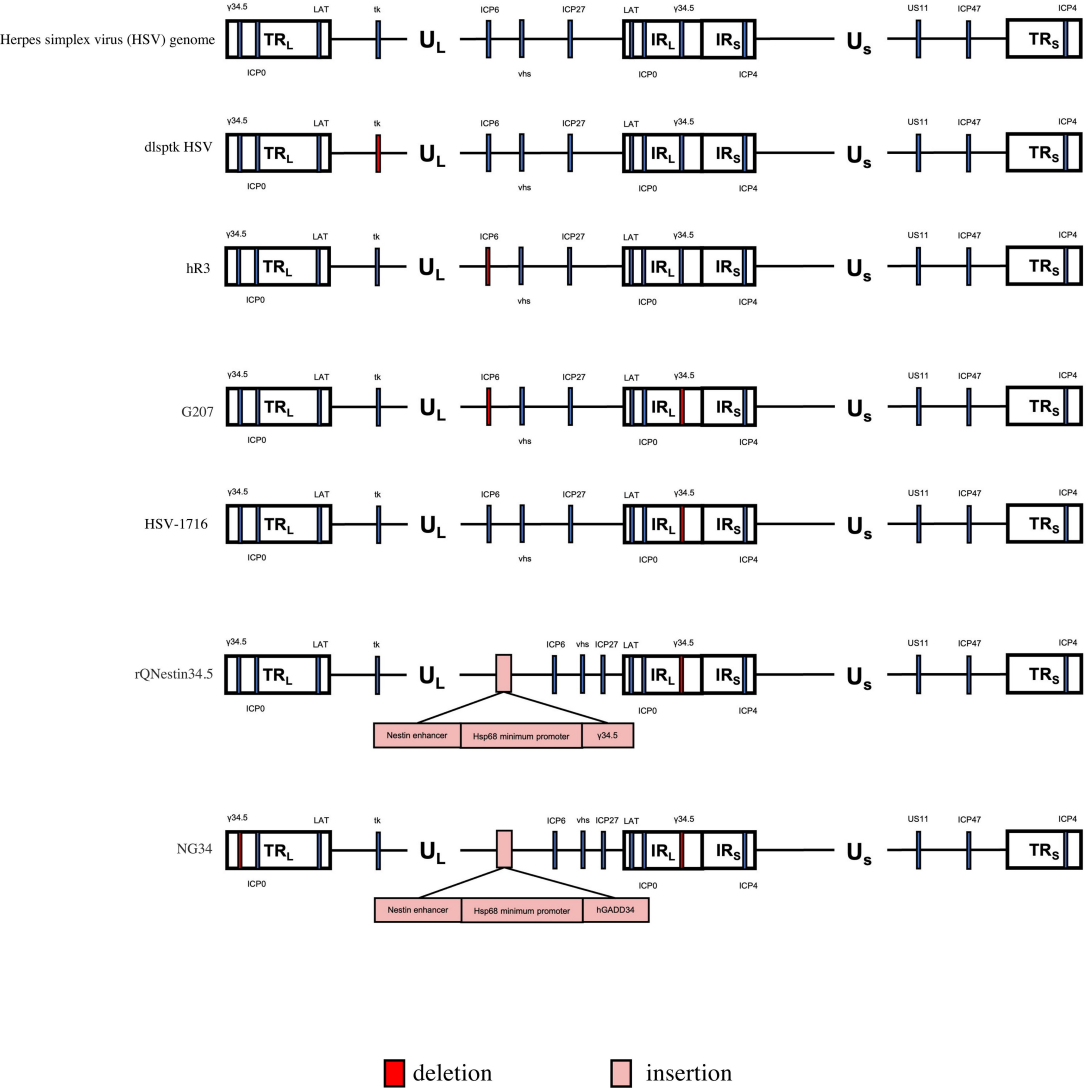
Genetic diagram of each generation of oncolytic HSV-1. The HSV-1 genome consists of long and short unique regions (UL and US) each bounded by terminal (T) and internal (I) repeat regions (R_L_, and R_s_). TK, thymidine kinase protein (TK). US11, unique short 11 gene. hGM-CSF, human granulocyte-macrophage colony stimulating factor gene. hGADD34, human homologue GADD34 replaces ICP34.5 for PP1 binding and eIF2a dephosphorylation. Nestin enhancer ,a glioma-specific enhancer.

HSV-1, an enveloped double-stranded DNA virus belonging to the Herpesviridae family, primarily infects and replicates in nervous tissue. Its antitumor activity was first observed in the 1950s when cancer patients with concurrent viral infections experienced tumor reduction (20). As a result, researchers explored different newly discovered viruses as potential cancer treatments. However, these therapies often caused significant toxicity to healthy tissues alongside tumor reduction, leading to a decline in the pursuit of viral-based cancer treatments (21). In 1989, Robert Martuza reported the inactivation of HSV-1 virus using the thymidine kinase gene. Subsequent treatment of glioma-bearing mice with this modified virus demonstrated no encephalitis. This discovery marked a turning point, making OVs a viable therapeutic option for GBM (22). To enhance safety and specificity, successive generations of OVs have been developed through genetic modification of the original HSV-1 wild type virus (**Figure 2**).

### 2.1 The first-generation oHSV lacking self-replication ability

#### 2.1.1 dlsptk HSV

The first modified version of herpes simplex virus (HSV), called dlsptk HSV, was developed in 1989 by Coen (23) and Martuza published the use of HSV dlsptk in 1991 (22). This engineered virus lacks the thymidine kinase protein (TK), which is necessary for viral replication in non-dividing cells. Studies by T. Valyi-Nagy showed that dlsptk HSV significantly extended the survival of mice with subcutaneous and orthotopic tumor models (24). However, it’s important to note that this modified virus still poses a risk of infection in immunocompromised patients. To ensure patient safety, alternative modifications of HSV-1 have been explored for tumor therapy.

#### 2.1.2 hrR3

The hrR3 mutant of HSV-1 was created by deleting ICP6 (UL39), the gene responsible for encoding the large subunit of viral ribonucleotide reductase. This enzyme plays a vital role in converting ribonucleotides to deoxyribonucleotides (dNMP) needed for viral genome synthesis. As a result, the absence of ICP6 in HSV-1 limits the availability of dNMP, thereby restricting the replication of hrR3 mutants to actively proliferating cells (25). Studies have shown that the mutant has considerable anti-cancer prospects. In previous studies, hrR3 showed a strong killing effect on human glioblastoma cell line cells, and in animal experiments, treatment with 5 × 10^6^ hrR3 plaque forming units showed a significant inhibitory effect on tumor growth (26). Experiments in which mutant herpes simplex virus 1 (hrR3) was injected into gliomas implanted in the brain of rats showed the lack of efficacy of hrR3 in the eradication of cancer due to interference of the immune system (27). As oHSV is further modified, safer and more effective oHSVs are made.

### 2.2 The second generation oHSV designed to target tumor cells with PKR-eIF2α mutations

The presence of the γ34.5 protein is crucial for assessing the neuropathogenicity of HSV. In normal cells, HSV infection triggers the phosphorylation of eIF2α by the cell’s protein kinase R (PKR), which prevents viral protein synthesis. However, the γ34.5 complex of HSV counteracts this process by dephosphorylating eIF2α, allowing wild-type HSV to replicate effectively in these cells. On the other hand, if γ34.5 is deleted from HSV, the modified virus loses its ability to replicate in normal cells. Interestingly, in cancer cells with a defective antiviral PKR-eIF2α pathway, deleting γ34.5 enables selective replication of HSV in these cells (28, 29). ICP6 is a large subunit of ribonucleotide reductase (RR) that is critical for viral replication and growth in nondividing cells. By deleting γ34.5 and inactivating ICP6, a safer second-generation oHSV was generated. Proliferation of the second-generation oHSV was restricted to tumor cells with PKR-eIF2α mutations.

#### 2.2.1 G207

G207 was generated by inserting the Escherichia coli lacZ gene into the coding sequence of viral ICP6 (UL39) and deleting two copies of the γ34.5 (RL1) locus in the viral genome (30). The ICP6 gene encodes the vital RR subunit essential for viral replication in non-dividing cells. Removing these genes restricts HSV-1 G207 proliferation to tumor cells (30). In preclinical animal models, the efficacy of G207 has been extensively demonstrated. Administration of 10^9^ plaque forming units (pfu) of G207 in BALB/c mice and Aotus nancymai owl monkeys’ brains showed no adverse effects (31). Remarkably, whereas 10^3^ pfu of wild-type HSV-1 proves lethal for Aotus nancymai owl monkeys, G207 has exhibited safety. Magnetic resonance imaging and histopathological evaluation of these primates revealed no central nervous system abnormalities, cell structure alterations, or presence of HSV immune response cells. These findings preliminarily verify the safety of G207 (32).

Markert et al. conducted a phase I clinical trial with 21 adults having advanced brain gliomas, demonstrating the safety of G207 without significant adverse effects. Tumor growth was suppressed in eight patients after one month of vaccination, and two patients achieved disease-free survival exceeding five years (33). These findings provide a strong basis for further clinical trials to explore the therapeutic potential of G207 in adults with recurrent brain gliomas. In another phase I trial implemented by James M. Markert et al., G207 combined with radiation therapy was investigated in nine patients with progressive, relapsed glioblastoma (34). The trial confirmed the safety of single-dose oncolytic HSV therapy augmented with radiation for treating malignant glioma patients. Despite observing reductions in tumor size and improved survival time, the absence of a control group necessitates additional clinical trials (NCT00157703) to establish the clinical therapeutic effect of G207.

G207 has demonstrated efficacy in treating adult patients with rGBM in multiple clinical trials. However, current research is mainly focused on assessing its effectiveness in pediatric patients with rGBM. Gregory K. Friedman conducted a phase I clinical trial (NCT02457845) to evaluate the safety, efficacy, and immune response of G207 in children with recurrent or progressive cerebellar brain tumors. The study aimed to enroll 15 participants and provide preliminary insights into using G207 to treat pediatric brain gliomas. The results showed that Intratumoral G207, alone or combined with radiation, had an acceptable adverse-event profile and exhibited evidence of responses in patients with recurrent or progressive high-grade glioma in pediatric cases. Additionally, G207 treatment converted immunologically “cold” tumors to “hot”. However, the loss of γ34.5 in G207 improved safety but impaired viral replication in glioblastoma stem cells (GSC) (35).

#### 2.2.2 HSV-1716

HSV-1716 is an example of an HSV variant that has undergone a 759-bp deletion in both copies of the ICP 34.5 gene, resulting in reduced neurotoxicity compared to the wild-type virus (36, 37). Additionally, the deletion of γ34.5 significantly limits the replication potential of HSV-1716 specifically in tumor cells.

HSV-1716 is worth noting that the antitumor effect of HSV-1716 is not solely attributed to its oncolytic activity but also to its direct anti-angiogenic properties. Through *in vitro* and *in vivo* experiments, Fabian Benencia et al. have confirmed the direct anti-angiogenic effects of the oHSV: HSV-1716 (38). One study demonstrates that HSV-1716 can specifically inhibit pediatric high-grade glioma (pHGG) and diffuse intrinsic pontine glioma (DIPG) migration and invasion, highlighting a novel mechanism of action for an OV against a principal hallmark of cancer. HSV1716 was also evaluated in this study, as it has previously been applied in early-phase trials for high-grade gliomas (39). Nine patients with high-grade glioma who had relapsed after curative treatment were treated with HSV-1716 injection to evaluate the safety of 1716 in patients with recurrent malignant glioma. The safety of HSV-1716 in the treatment of gliomas was demonstrated without toxic effects at intratumoral doses up to 10^5^ p.f.u (40).. Another study involved 12 patients with rGBM who received tumor injections of 10^5^ HSV1716 p.f.u. Viral replication and an immune response to HSV1716 were detected after vaccination, proving that HSV1716 replicates in rGBM and does not trigger a toxic reaction in the patient (41). S Harrowet al. enrolled 12 patients with advanced gliomas for intratumoral injection of HSV1716 to observe the treatment of HSV1716. The results showed a promising improvement in the survival of GBM patients after HSV1716 inoculation compared to the expected median survival of GBM patients (42). An additional clinical trial of HSV-1716 for glioma included two patients with recurrent pediatric glioma who underwent surgical resection. However, the results of this trial (NCT02031965) have not yet been reported.

#### 2.2.3 rQNestin34.5 and NG34

Deletion of two copies of γ34.5 in HSV restricts its replication in tumor cells, albeit with a significant reduction in overall replicative capacity. To address this issue, Hiromasa Kambara et al. developed rQNestin34.5, a novel selective mutant of HSV-1 (43). This mutant reintroduces a single copy of γ1 34.5 into the viral genome under the control of the nestin gene enhancer (a glioma-specific enhancer) and the hsp68 promoter.*In vitro* and *in vivo* experiments demonstrated robust replication and oncolytic activity of rQNestin34.5 specifically in glioma cells (44). Safety evaluations conducted in immunized and immunodeficient mice further supported its safety profile (43). Notably, the Dana-Farber Cancer Institute is currently sponsoring a Phase I clinical trial (NCT03152318) investigating the therapeutic potential of rQnestine 34.5V. 2, a transgenic HSV-1 virus, in recurrent glioblastoma alongside cyclophosphamide-based immunoregulation.

In the absence of active nestin enhancer, transcriptional leakage and minimal functionality from the hsp68 promoter in normal CNS neuronal cells may generate a small amount of ICP34.5, potentially leading to neurotoxicity induced by rQNestin34.5 (44, 45). NG34, an enhanced version of rQNestin34.5, employs GADD34 as a substitute for ICP34.5 to bind PP1 and dephosphorylate eIF2α (46). This replacement eliminates the beclin-1 binding motif responsible for neurotoxicity and autophagy inhibition in ICP34.5 (47). In human *in situ* GBM mice, NG34 demonstrates comparable efficacy to QNestin34.5 while exhibiting reduced neurotoxicity.

Tumor cells commonly express PD-1 to suppress immune cell destruction (48, 49). Intra-tumor administration of an oHSV carrying a scFv (single chain fragment variable) antibody against PD-1 can enhance the anti-tumor immune response without compromising the virus’s oncolytic efficacy. NG34scFvPD-1, obtained by modifying NG34 with CMV-controlled scFvPD-1 cDNA, has shown expression of a single-chain antibody against mouse PD-1 in animal experiments. NG34scFvPD-1 exhibits comparable oncolytic properties to NG34 *in vitro* and improved survival rates in immunoactive mice. Furthermore, immunocompetent mice develop anti-tumor immune memory, protecting against tumor metastasis (50).

#### 2.2.4 G47Δ

The G47Δ vector, a modified version of HSV-1 based on the G207 vector, was constructed by further deleting the α47 gene. Because the expression of the α47 gene inhibits the antigen presenting (TAP) associated transporter, this deletion leads to increased MHC class I expression in infected cells, resulting in enhanced activation against tumor T cells. Additionally, G47Δ incorporates the late US11 gene under the control of the early α47 promoter, effectively suppressing the growth properties of the γ34.5 deficient mutant (51). It is noteworthy that G47Δ has obtained approval in Japan for glioblastoma treatment.

The University of Tokyo conducted a clinical study (UMIN-CTR: UMIN000002661) on G47Δ in patients with recurrent brain gliomas for the first time, specifically glioblastoma (52). This open-label, single-armed phase I-II trial aimed to assess the safety of intracranial administration of G47Δ. Subsequently, IMSUT Hospital initiated a Phase II clinical trial (UMIN-CTR: UMIN000015995) to evaluate the effectiveness and safety of G47Δ in patients with residual or recurrent glioblastoma who had previously received radiation therapy and TMZ chemotherapy. The trial involved intratumoral and repeated administration of G47Δ in 19 adult patients with rGBM. After excluding those with IDH1 mutations, among the remaining 13 patients, the one-year survival rate after G47Δ initiation was 84.2%, with a median OS of 20.2 months and a median PFS of 4.7 months. In comparison, treatment with chemotherapeutic agents resulted in a median OS of 5.0 months and a median PFS of 1.8 months. G47Δ demonstrated superior survival benefits and a favorable safety profile for treating rGBM (53). These pivotal trial findings led to the conditional and time-limited approval of G47Δ for GBM by the Ministry of Health, Labour and Welfare (MHLW) on June 11, 2021, positioning G47Δ as a potential breakthrough in glioblastoma treatment, offering improved survival outcomes and the possibility of a cure for a subset of patients. It may become the first new drug since TMZ and the first new treatment since TTF.

G47Δ is currently undergoing preclinical and clinical studies for stomach cancer, prostate cancer, and other types of cancer, where it has demonstrated superior anti-tumor efficacy compared to G207 and excellent safety characteristics. At lower multiple infection rates, G47Δ exhibits enhanced killing effects in human prostate cancer cell lines LNCaP and DU145, resulting in a 22-fold increase in viral production. Treatment with G47Δ in a mouse model of prostate cancer reduces tumor growth in established s.c. TRAMP and HONDA tumors, as well as inhibiting the recurrence of HONDA tumors previously regressed by androgen ablation therapy (54).

Furthermore, G47Δ shows promising therapeutic potential for human gastric cancer. *In vitro* experiments confirm favorable cytopathic effects and replication of G47Δ in nine tested human gastric cancer cell lines. *In vivo* intratumoral inoculation of G47Δ (at 2×10^5^ or 1×10^6^ pfu) significantly suppresses subcutaneous tumor growth in MKN45, MKN74, and 44As3 models (55).

In summary, these findings indicate that G47Δ may possess potent inhibitory effects on various tumor types beyond brain gliomas.

## 3 Adenovirus -based oncolytic viruses

Oncolytic adenovirus (also known as conditionally replicating adenovirus or CRAd) is a natural selection or genetically engineered adenovirus. Utilizing the distinguishing characteristics of tumor cells and normal cells in terms of structure and metabolic pathways, oncolytic adenovirus selectively proliferates and replicates within tumor cells, resulting in their lysis. Specifically, wild-type adenovirus has been enhanced to replicate within tumor cells and effectively lyse the target cells while minimizing its toxicity toward normal cells. At present, the third-generation of adenovirus modification for glioma treatment has been achieved. Genetically engineered or screened conditionally replicating viruses utilize aberrant molecular/genetic pathways within tumors, ensuring non-toxicity in normal cells. They are designed to replicate efficiently solely within cancer cells, leading to the lysis and destruction of the infected cancer cells (56).

**Figure 3:**
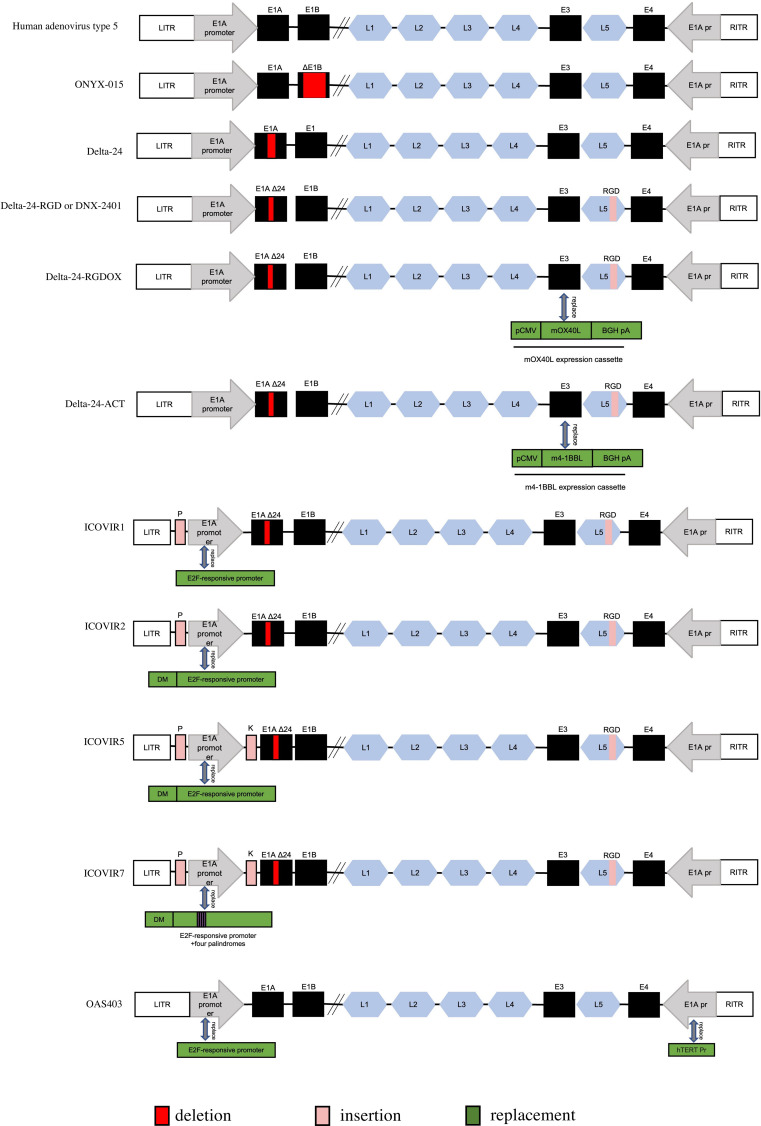
Genetic diagram of each generation of Oncolytic adenovirus. ITR, inverted terminal repeat. E1AΔ24: a deletion of 24 base pairs within the E1A region. DM, insulator DM-1. E2Fp, E2F-responsive promoter. K, a Kozak sequence. P, E2F-responsive palindromes (8 E2F-binding sites). RGD, an RGD integrin-binding motif in the HI loop of the fiber. pCMV, the cytomegalovirus promoter. mOX40L, mouse OX40L cDNA. BGH PA, bovine growth hormone poly-adenylation signal. The mOX40L expression cassette replaces the E3 region in Delta-24-RGDOX. hTERT Pr, human telomerase reverse transcriptase promoter.

Adenovirus is a nonenveloped virus with icosahedral capsid containing a 36kD double-stranded linear genome. The genome of the virus can be categorized into two distinct regions: the early gene region (E1-E4) and the late gene region. The former primarily governs viral replication and transcriptional regulation, while the latter plays a pivotal role in the synthesis of structural proteins (57). Within the early gene region, viral regulatory proteins are encoded, which play a crucial role in controlling the expression of late genes. Notably, the initial expression of E2 products, including those stemming from the E1 gene, is essential for adenovirus genome replication, virus packaging, and protein translation processes (58). Due to this significance, current genetic modification strategies for adenovirus primarily concentrate on targeting the E1 region (**Figure 3**).

### 3.1 The first-generation CRAds specifically targeting p53 mutated cancer cells

The wild-type p53 protein acts as a tumor suppressor, preventing abnormal cell proliferation and eliminating cells with abnormalities. Mutated p53, on the other hand, loses its regulatory function in controlling the normal cell cycle (59, 60). GBM is an aggressive brain cancer with a poor prognosis, and the frequency of p53 mutations varies between primary and secondary GBM. Approximately 30% of primary cases and 65% of secondary cases exhibit p53 mutations (61). In wild-type adenovirus infection, p53-mediated apoptosis is activated during the S phase. However, adenovirus E1B protein inactivates p53, inhibiting p53-mediated apoptosis and promoting viral replication (62). To address this, the first conditional adenovirus was developed by introducing E1B deficiency, exemplified by ONYX-015.

#### 3.1.1 ONYX-015

ONYX-015 is a genetically modified CRAd derived from a human type 2/5 chimeric adenovirus with an E1B deletion. This modification allows ONYX-015 to selectively replicate in cancer cells lacking functional p53 while sparing normal cells with intact p53 (63–65). Initial reports indicated that ONYX-015 selectively killed p53-deficient tumor cells (62) ^(^ 66 ^),^. The NABTT CNS Consortium conducted a Phase I trial to evaluate the efficacy and safety of ONYX-015 in recurrent brain gliomas. Twenty-four adult patients with recurrent gliomas received multiple injections of ONYX-015 at the margins of resected recurrent gliomas. The median time to progression after treatment was 46 days, and the median survival time was 6.2 months. No serious adverse events occurred in any of the 24 patients, validating the therapeutic safety of ONYX-015. The NABTT CNS Consortium conducted a Phase I trial to evaluate the efficacy and safety of ONYX-015 in recurrent brain gliomas. Twenty-four adult patients with recurrent gliomas received multiple injections of ONYX-015 at the margins of resected recurrent gliomas. The median time to progression after treatment was 46 days, and the median survival time was 6.2 months. No serious adverse events occurred in any of the 24 patients, validating the therapeutic safety of ONYX-015 (67). However, ONYX-015 did not exhibit significant antitumor effects on recurrent gliomas, suggesting that p53 may not play a key role in the tumor selectivity of ONYX-015. A Phase II clinical trial combining intratumor ONYX-015 therapy with standard intravenous cisplatin and 5-fluorouracil (5-FU) chemotherapy in patients with recurrent squamous cell carcinoma of the head and neck yielded promising results, with tumors not progressing at 6 months in the combination group (68).

Recent studies have shown that ONYX-015 replication is not strictly restricted to p53-mutated tumor cells and can replicate and eliminate cells even when the p53 pathway is intact (64, 65) ^(^ 69–72 ^),^. The tumor selectivity of ONYX-015 relies not only on the p53 protein but also on the RNA export function of E1B-55K provided by tumor cells. Additionally, ONYX-015 exhibits limited replication and toxicity in tumor cells without p53 mutations (68). Despite these findings, ONYX-015 currently does not have any registered clinical trials in the United States following the withdrawal of a Phase I trial by the Dana-Farber Cancer Institute in 2013.

### 3.2 The second-generation CRAd specifically targeting cancer cells with mutations in the Rb pathway

ONYX-015’s limited progress in clinical trials is attributed to its untargeted viral replication and inability to infect CAR (Coxsackie adenovirus receptor)-deficient tumor cells. However, the presence of multiple adenovirus genes targeting cell cycle regulators provides an opportunity to develop oncolytic adenoviruses that can target alternative pathways. Since abnormalities in the p16/Rb/E2F pathway are present in most gliomas, viral therapies targeting the Rb pathway were developed as the second-generation of CRAd (73).

#### 3.2.1 DNX-2401

A typical example of a second-generation CRAd targeting the Rb pathway is the Delta-24 adenovirus derived from human adenovirus type 5. Delta-24 carries a 24-base pair deletion in the Rb-binding domain of the E1A gene, resulting in a mutant E1A (mE1A) protein. Normally, the E1A protein binds to the Rb protein, releasing E2F and allowing cell progression into the S phase. In Delta-24, however, mE1A fails to effectively bind the Rb protein, leading to limited E2F release. As a result, Delta-24 cannot replicate in normal cells since mE1A is unable to bind the Rb protein and release E2F (74). Notably, Stolarek, R et al. demonstrated that Delta-24 exhibits replicative capability and cytotoxicity against medulloblastoma cells (75).

Delta-24 has demonstrated the ability to sensitize glioma cells to the camptothecin analogue irinotecan (CPT-11), both *in vitro* and *in vivo*. This effect is achieved through the upregulation of topoisomerase I expression and the induction of cancer cell accumulation in the S phase. The sequential administration of Delta-24 and CPT-11 has shown a significant extension in the survival time of animals with glioma. Therefore, the combination of adenovirus therapy and chemotherapy enhances its anticancer effect (76).

To enter host cells, Delta-24 first binds to the coxsackievirus and adenovirus receptor (CAR) on the cell surface. However, certain cancers, including glioblastoma, exhibit low levels of CAR, which greatly limits the infectivity of Delta-24. The internalization of adenovirus into host cells is facilitated by secondary interactions between the RGD motif on the Penton-based protein ring and integrins (αvβ5 and αvβ3) (77). In order to address this limitation, the gene encoding the arginine-glycine aspartic acid (RGD) peptide was introduced into the viral fiber knob receptor of Delta-24, resulting in the second-generation Delta-24-RGD or DNX-2401. Integrins are commonly overexpressed on cancer cells (78). Consequently, the infection rate of Delta-24-RGD in glioblastoma was significantly increased (79, 80).

Lang et al. conducted a Phase I dose escalation trial of DNX-2401 in 37 patients with rGBM. Group A (n=25) received eight dose levels of DNX-2401 via a single intratumoral injection to assess safety and reactivity, while Group B underwent intratumoral injection using a permanently implanted catheter, followed by en bloc resection after 14 days to obtain post-treatment specimens. The results revealed that 20% of patients in Group A experienced survival beyond three years, with at least three patients exhibiting over 95% reduction in enhanced tumor survival, resulting in more than three years of PFS. Evaluation of post-treatment specimens demonstrated virus replication and spread within the tumor in Group B, indicating direct virus-induced oncolysis. These clinical trial findings suggest that DNX-2401 can achieve prolonged survival through its direct oncolytic effect and induction of immune-mediated anti-glioma response (NCT00805376) (81). These observations agreed with preclinical studies showing that Delta-24-RGD infection induces autophagy and immunogenic cell death in glioblastoma (82, 83).

In a pre-clinical study by Martinez-Velez N, the anti-glioma effects of Delta-24-RGD were assessed in pediatric pHGG and DIPGs models. The experimental data indicated significant antitumor effects of Delta-24-RGD in both cell lines and mouse models of pHGG and DIPG. Additionally, Delta-24-RGD administration triggered an anti-tumor immune response alongside its oncolytic effects. These promising preclinical findings lay the foundation for a Phase I/II clinical trial investigating DNX-2401 (NCT03178032) (84).

#### 3.2.2 DNX-2401 and chemotherapy

In 2017, a Phase I trial (NCT01956734) evaluated the combination of DNX-2401 and TMZ for treating rGBM. Likewise, Clinica Universidad de Navarra conducted a Phase 1b, randomized, multicenter, open-label study (TARGET-I, NCT02197169) from 2014 to 2018, investigating conditional replicative adenovirus (DNX-2401) and interferon gamma (IFN-γ) for recurrent glioblastoma. However, no published results are available from these trials, necessitating additional clinical trials to demonstrate DNX-2401’s efficacy in treating rGBM.

In a completed Phase II trial (NCT02798406), the efficacy and safety of DNX-2401 in combination with Pembrolizumab for treating rGBM were investigated. The study included 49 glioma patients who received intratumoral treatment with various doses of DNX-2401 viral particles (vp) (5*10^8^ vp, 5*10^9^ vp, and 5*10^8^ vp DNX-2401) alongside Pembrolizumab. The median OS was 12.5 months (10.7 to 13.5 months). Additionally, 56.2% (95%CI 41.1-70.5%) of patients achieved clinical benefit, defined as disease stabilization or improvement. Importantly, no toxic effects were observed with DNX-2401, even at a maximum dose of 5*10^10^ vp. These findings demonstrate the safety and significant survival benefits of combining DNX-2401 and Pembrolizumab for selected patients with recurrent brain gliomas (85). Clinical trials have shown a survival benefit when using DNX-2401 in combination with chemotherapy. However, it is worth noting that these trials lacked a negative control group that used chemotherapy alone. Additional clinical trials are required to confirm whether the combination of DNX-2401 and chemotherapy can indeed extend patient survival.

#### 3.2.3 DNX-2401 and radiotherapy

Radiotherapy (RT) is commonly used in managing gliomas, but its effectiveness is limited to temporary clinical improvements. Thus, researchers evaluated the feasibility, safety, and efficacy of combining Delta-24-RGD with RT for pHGGs and DIPGs. This combination significantly improved survival rates and increased immune cell infiltration at the tumor site in treated mice (86). These promising findings suggest the potential of Delta-24-RGD and RT combination therapy for clinical use in pHGGs and DIPGs.

#### 3.2.4 Delta-24-ACT

Delta-24-ACT, a modified oncolytic adenovirus, incorporates the 4-1BB ligand (4-1BBL) to enhance its therapeutic capabilities. Infected glioma cells express elevated levels of 4-1BB ligand, which binds to TNFRSF9 (CD137; 4-1BB), a co-stimulatory receptor. This interaction activates T cells and immune cells, augmenting the oncolytic effect of the adenovirus. The antitumor effects and induction of T cell activation by Delta-24-ACT have been validated in glioma cell lines. In CT-2A tumor-bearing glioma mouse models, both Delta-24-RGD and Delta-24-ACT improved median survival, with Delta-24-ACT exhibiting slightly superior efficacy over Delta-24-RGD treatment (87). Preclinical models have demonstrated the potent antitumor effects of 4-1BB agonists. However, in clinical trials, their use in cancer treatment has been hindered by notable hepatotoxicity (88, 89). To overcome this challenge, one potential approach is specifically targeting Delta-24-ACT to tumor cells. This allows bypassing the systemic administration of 4-1BB agonists and improving safety. By delivering Delta-24-ACT directly into the tumor, it can disrupt microenvironment tolerance observed in DIPG, triggering proinflammatory changes that activate T cells and generate immune memory (90). The safety and preclinical efficacy of Delta-24-ACT have been well-established. Yet, further clinical trials are necessary to evaluate its oncolytic effect and induction of anti-tumor immune response, as it represents a potential novel oncolytic virus.

#### 3.2.5 Delta-24-RGDOX (DNX-2440)

Delta-24-RGDOX (DNX-2440), an improved version of Delta-24-RGD, stimulates immunostimulating OX40 ligand (OX40L) expression on infected tumor cells, activating T cells recognizing tumor cell antigens. In immunologically competent mouse glioma models, Delta-24-RGDOX induces more effective *in situ* autologous cancer vaccination than Delta-24-RGD, resulting in a lasting tumor-specific therapeutic effect (91). Currently, an ongoing Phase I clinical trial (NCT03714334) at Clinica Universidad de Navarra investigates stereotactic injection therapy using OVs DNX-2440 for patients experiencing their first or second recurrence of GBM.

### 3.3 The third-generation CRAds utilizing the human E2F-1 promoter

Transcription factors of the E2F family play an important role in entry into the S phase of the cell cycle (92, 93). E2F function is inhibited upon binding to the retinoblastoma tumor suppressor protein (pRb). Binding of E2F factors to nonphosphorylated pRb prevents E2F-mediated transactivation, but this complex also actively represses transcription when bound to the promoter. It is thought that all RB pathways in tumors have changes that inhibit the binding of pRb to E2F, which leads to an increase in free E2F (94, 95). the third-generation CRAds were obtained by replacing The E1A promoter of the second-generation CRAds with the human E2F-1 promoter. E2F-1 promoter can selectively replicate. The third-generation CRAds in tumor cells and reduce hepatotoxicity.

#### 3.3.1 ICOVIR-1、ICOVIR-2、ICOVIR-5

To enhance adenovirus selectivity for glioma cancer cells, Majem et al. replaced the native E1A promoter in Delta-24-RGD with the human E2F-1 promoter, resulting in ICOVIR-1 (96). They further introduced the DM-1 element upstream of the E2F-1 promoter to create ICOVIR-2, which acted as an enhancer blocking insulator to reduce activity against normal cells. Cell experiments demonstrated that both ICOVIR-1 and ICOVIR-2 effectively prevented E1A expression in normal human cells, leading to reduced viral replication (97).

Based on ICOVIR-2, ICOVIR-5 was optimized by inserting the CCACC sequence (Kozak sequence) before the first codon of the E1a gene. This alteration aimed to enhance transcription of the heteroe2F-1 promoter (98). In 2013, Garcia et al. conducted a Phase I clinical trial with injectable ICOVIR-5 in 14 melanoma patients. While ICOVIR-5 could reach melanoma metastases after single intravenous administration, tumor regression was not observed in the evaluated patients. These findings support ICOVIR-5’s potential for treating disseminated cancer. Currently, no clinical trials are investigating ICOVIR-5 for glioma treatment (99). Nevertheless, as a safer and more potent conditional adenovirus, ICOVIR-5 holds significant promise for clinical applications in glioma treatment.

#### 3.3.2 OAS403

The oncolytic adenovirus OAS403 utilizes a human adenovirus type 5 vector with the incorporation of the E2F-1 promoter. This promoter regulates the early region E1A in OAS403, while a human telomerase reverse transcriptase promoter controls the E4 region, which encodes toxic viral proteins responsible for cell damage (100). The inclusion of the human telomerase reverse transcriptase promoter in the E4 region allows selective expression of these toxic proteins specifically in cancer cells (101, 102). In a mouse tumor model, a single intravenous injection of OAS403 at a dosage of 3×10^12^ vp/kg showed significant antitumor efficacy. Particularly, in a preestablished LNCaP prostate tumor model, systemic administration of OAS403 resulted in complete tumor regression in over 80% of cases at tolerable doses. Moreover, an increase in site-specific viral replication within the tumor was observed, with no discernible growth in the liver. Additionally, combining OAS403 treatment with Adriamycin significantly enhanced its efficacy (103). OAS403 shows promise for treating various human cancers, including recurrent glioma. An alternative variant, ICOVIR-7, incorporates an additional E2F response site palindrome within the insulated E2F-1 promoter to exert greater control over E1A-δ24 and enhance E2F-dependent E1A gene expression (104).

## 4 Other oncolytic viruses

In addition to oHSV and CRAd, various other OVs such as reovirus, poliovirus, and retrovirus have been modified and explored for their potential in treating brain glioma. Notably, Pelareore (reovirus), PVSRIPO (poliovirus), and Toca 511 (retrovirus) have made significant clinical advancements.

### 4.1 Pelareore

Reovirus is a non-encapsulated icosahedral double-stranded RNA virus that selectively targets cells with activated Ras signaling pathways. It has therapeutic potential for various solid and hematological tumors, including pancreatic, colorectal, thyroid, and lung cancers, as well as acute myeloid leukemia (105–107). REOLYSIN^®^ (Pelareore), derived from reovirus strain type 3 Dearing virus, is an FDA-designated fast track treatment for metastatic breast cancer and metastatic pancreatic ductal adenocarcinoma. Safety of reovirus has been demonstrated in two Phase I trials involving adult brain tumor patients, without reaching the MTD (108, 109). Studies on mice have shown the efficacy of the GM-CSF (sargramostim)/intravenous REOLYSIN^®^ regimen for brain glioma (110). A Phase I trial on six pediatric patients with recurrent or refractory advanced brain tumors has explored the combination of GM-CSF and Pelareore, but the MTD remains undetermined (NCT02444546). Regrettably, despite the induction of an immune response in patients using the combination of GM-CSF and pelareoreep, no complete or partial tumor response was observed. Instead, all patients experienced disease progression within 60 days (111). The limited clinical efficacy of pelareore in glioma may be attributed to factors like the blood-brain barrier and immune clearance. To optimize the antitumor effect of pelareore, future clinical trials could explore the use of multiple para-tumor injections in combination with GM-CSF. This approach shows potential for enhancing the therapeutic outcomes of pelareore and requires further investigation.

### 4.2 Recombinant nonpathogenic polio-rhinovirus chimera (PVSRIPO)

PVSRIPO, also known as lerapolturev, is a modified version of poliovirus type 1 (Mahoney), where the internal ribosome entry site is replaced with the human rhinovirus type 2 I.E. element. While maintaining its affinity for the CD155 receptor, PVSRIPO exhibits reduced virulence compared to the wild-type poliovirus (112, 113). The FDA recognized its potential and granted PVSRIPO breakthrough therapy designation in 2016. The efficacy of PVSRIPO has been confirmed in a Phase I clinical trial involving 61 adult patients with recurrent WHO grade IV malignant glioma. In this trial, intratuminal infusion of PVSRIPO established a safe dose (3.3×10^9^ TCID50) for direct delivery to intracranial tumors. The results showed significantly higher OS rates at 24 and 36 months in the PVSRIPO immunotherapy group compared to historical controls (21% vs 14% at 24 months; 21% vs 4% at 36 months) (NCT01491893) (114). The median survival in children with recurrent glioma is typically less than 6 months. However, a Phase I clinical trial administered polio-rhinovirus chimera lerapolturev to patients, resulting in one patient (1/8) surviving beyond 22 months. This finding suggests the potential of PVSRIPO (poliovirus) to prolong the lifespan of rGBM. Nonetheless, further validation through extensive studies is necessary to affirm these trial results conclusively (115). Building on this progress, Duke University is currently conducting a Phase II clinical study involving 122 adult patients with glioblastoma. The study aims to investigate the safety, efficacy (antitumor response, and survival) of PVSRIPO (NCT02986178). Moreover, preclinical studies have shown that combining PVSRIPO with immune checkpoint inhibitors can effectively target tumors. Consequently, a Phase II trial combining PVSRIPO and the immune checkpoint inhibitor Pembrolizumab is underway, involving 30 patients with recurrent glioblastoma (NCT04479241).

### 4.3 Toca 511

Toca 511 is a modified non-soluble mouse leukemia virus vector that incorporates the cytosine deaminase (CD) enzyme gene, allowing for selective infection of tumor cells (116–119). Selective infection of tumor cells by Toca 511 results in the expression of the CD enzyme, which facilitates the conversion of the prodrug 5-fluorocytosine (Toca FC) into 5-FU within these cells, enabling targeted chemotherapy (120, 121). Toca 511 causes direct cytotoxicity and proinflammatory state of cancer cells via 5-FU. In a Phase I clinical study (NCT01470794), 43 patients with recurrent glioblastoma were treated with Toca 511, leading to a median survival of 13.6 months, compared to 7.1 months for untreated patients (122). Two additional Phase I studies (NCT01156584 and NCT01985256) demonstrated that intratumoral and intravenous administrations of Toca 511 as standalone treatments for rHGG is safe and tolerable.

However, a Phase III clinical trial (NCT02414165) involving 403 patients with recurrent glioblastoma and anaplastic astrocytoma treated with Toca 511/FC did not demonstrate a significant advantage over the standard of care (SOC) group. The Toca 511/FC group showed a median survival of 11.1 months, while the SOC group exhibited a median survival of 12.22 months (122). Further clinical trials are needed to provide robust evidence regarding the efficacy of Toca 511/FC in the treatment of recurrent glioma.

### 4.4 Zika virus

Zika virus (ZIKV) belongs to the Flaviviridae family and is an RNA virus. Its viral genome encodes a single polyprotein through a sole open reading frame, subsequently cleaved by cellular and viral proteases into ten proteins (123). Since 2015, ZIKV infection in pregnant women has emerged as a global public health emergency due to its association with microcephaly and other congenital diseases (124). Recent studies have identified ZIKV’s specific targeting of GSCs and its oncolytic activity (125). Moreover, ZIKV has been found to participate in viral endocytosis mediated by SOX2 and integrin avb5, which play roles in immune response suppression, GBM progression, and GSC maintenance (126–128). Notably, integrin avb5, typically expressed at low levels in normal tissues, exhibits heightened expression in tumors (128). Consequently, ZIKV has garnered attention as a potential oncolytic virus for treating GBM.

A safer live-attenuated Zika virus vaccine, ZIKV-LAV, was developed to enhance sensitivity to the host’s innate immune response. This vaccine is characterized by a 10-nucleotide deletion in the 3’ untranslated region (3’UTR) of the viral genome (129). Administration of ZIKV-LAV via brain injection in mice showed no detectable behavioral abnormalities, neurovirulence, or organ damage, confirming its high safety profile. Furthermore, ZIKV-LAV-treated mice exhibited significantly longer median survival times compared to the mock group (130, 131).

Contrarily, glioma slice cultures exhibit resistance to Zika virus infection unlike NPC, which is attributed to interferon-beta secretion by myeloid cells in the glioblastoma tumor microenvironment (132, 133). The combination of CDK4/6 inhibitors with ZIKV-LAV enhances the selective replication of the vaccine, resulting in significant inhibition of tumor growth and prolonged survival in glioma mice. Although ZIKV-LAV is currently in the preclinical stage, the combination of ZIKV-LAV with immunosuppressants shows promise as a novel immunotherapy for glioma.

## 5 Discussion

### 5.1 Current status

In the past 20 years, oncolytic viruses have achieved exciting results in the treatment of glioma. OVs have displayed promising results in the treatment of glioma over the past two decades, thanks to the development and application of genetic engineering technologies. These advancements have rendered OVs more specific, effective, and safe. Numerous oncolytic virus studies are currently undergoing phase I, II, and III clinical trials. While the first-generation of OVs, including dlsptk HSV and ONYX-015, demonstrated promise in preclinical trials, they were ultimately eliminated due to safety concerns and non-significant oncolytic effects during clinical trials. Subsequent modifications gave rise to second/third-generation oHSV and CRad, which exhibit great potential in treating glioma. Notably, DNX 2401 combined with PD-1 significantly prolonged the survival time of patients with glioma, and G47Δ has received approval for glioma treatment in Japan. Furthermore, ICOVIR-7 has shown promise in preclinical trials by displaying lower toxicity and increased antitumor efficacy compared to ICOVIR-5 in a subcutaneous xenograft mouse model (134). A comprehensive summary of ongoing and completed human trials investigating OVs in glioma can be found in **Table 1**. Despite the favorable progress achieved in most current OVs clinical trials, several limitations persist. These trials often impose strict inclusion criteria driven primarily by safety concerns, resulting in a limited patient population and random trial outcomes. Moreover, the evaluation of OVs efficacy and potential long-term consequences in GBM remains inadequate. The reliance on historical survival as a control further emphasizes the necessity for prospective randomized trials to effectively assess the effectiveness of OVs.

### 5.2 Future directions

To ensure the consistency between preclinical and clinical test results, conducting animal experiments is crucial for evaluating the safety and effectiveness of OVs-related products prior to clinical trials. However, it should be noted that the antitumor effect of OVs is partially mediated by inducing an immune response against the virus. Therefore, using immunodeficient mice in the PDX model to evaluate the oncolytic effects of OVs on brain gliomas disregards the impact of the immune response. Utilizing an induced glioma model can provide a more comprehensive assessment of OV’s therapeutic efficacy.

Despite promising results in preclinical and clinical trials, OVs have not yet demonstrated improved patient outcomes compared to standard care modalities like surgery, radiotherapy, and chemotherapy. This lack of improvement can be attributed to factors such as tumor heterogeneity, immune evasion, therapy resistance, limitations of the blood-brain barrier (BBB), and TME. To enhance therapeutic efficacy, it is recommended to classify OVs based on specific brain glioma types and target the p53 and Rb pathways according to their respective mutation types.

Numerous studies have investigated different routes of administration for OVs in glioma treatment, including intravenous injection, arterial injection, inhalation, intratumoral injection, and convection-enhanced delivery (CED). Intratumoral injection and CED are preferred due to their ability to provide local drug delivery to tumor lesions without opening the blood-brain barrier, although surgery is required (135). Alternatively, direct systemic administration can utilize adjunctive measures to actively open the blood-brain barrier, avoiding surgery (136). Developing new delivery routes that penetrate the blood-brain barrier and target tumors precisely is critical. Nanoparticles or cell-based carriers present non-invasive alternatives for patients who cannot undergo neurosurgical procedures. Improved imaging models are clinically needed to assess patient responses to OVs treatment and guide subsequent therapy. Additionally, determining the maximum safe dose of each OV is essential to maximize killing effect while ensuring patient safety.

The immune response, responsible for clearing OVs and hindering their replication, is a major reason for the effectiveness of oncolytic virotherapy. Combining OVs with immunosuppressants, such as anti-PD1 antibodies, has shown promise in clinical trials (137). Achieving a balance between immune response and clearance through the use of immunosuppressants and multi-dose OVs delivery requires further investigation. Combining oncolytic virotherapy with T cell therapy can help proliferate T cells in the local tumor microenvironment for optimal efficacy. Further understanding of immune mechanisms can aid in the development of improved OVs and expand their potential.

### 5.3 Conclusions and perspective

Recurrent gliomas, grade 4 tumors with a poor prognosis, do not see significant improvement in survival rates despite standard treatments like surgery, radiation therapy, and TMZ chemotherapy (138). New treatment methods are therefore necessary to improve patient outcomes.

One such promising approach is oncolytic virus therapy. This viral genome-based treatment selectively replicates in tumor cells while targeting them specifically, making it a hopeful treatment option for patients with recurrent tumors. Advancements in oncolytic virus genome modification have led to improvements in safety and anticancer efficacy. First-generation OVs include dlsptk HSV and ONYX-015, while second-and third-generation OVs consist mainly of oHSV and CRAd, respectively. Although recurrent glioma has a high mortality rate and poor prognosis, oncolytic virus therapy shows promise in improving patient outcomes due to its favorable safety profile. By directly targeting glioma cells and inducing cell death through selective replication and immune stimulation via acting as antigens, oncolytic viruses hold potential as a treatment option. However, safety and efficacy concerns remain, emphasizing the importance of developing safer and more effective oncolytic virus vectors. Additionally, optimizing virus delivery routes, enhancing specificity to tumor cells, limiting antiviral responses, enhancing anti-tumor immunity, reprogramming and reshaping the tumor immune microenvironment, and identifying drugs with similar anticancer effects are viable ways to improve oncolytic virus therapy.

## Author contributions

MH: Data curation, Formal Analysis, Methodology, Writing – original draft, Writing – review & editing. XL: Methodology, Writing – review & editing. YT: Data curation, Formal Analysis, Writing – original draft. YC: Funding acquisition, Supervision, Writing – review & editing.

## Glossary

**Table:**

GBM	Glioblastomas
WHO	The World Health Organization
TMZ	Temozolomide
rGBM	Recurrent glioblastoma
mOS	Median overall survival
OV	Oncolytic virus
HSV-1	Herpes simplex virus-1
Ad	Adenovirus
NDV	Newcastle disease virus
RV	Reovirus
oHSV	Oncolytic herpes simplex virus
TK	Thymidine kinase protein
dNMP	Deoxyribonucleotides
PKR	Protein kinase R
RR	Ribonucleotide reductase
pfu	Plaque forming units
GSC	Glioblastoma stem cells
pHGG	Pediatric high-grade glioma
DIPG	Diffuse intrinsic pontine glioma
scFv	Single chain fragment variable
TAP	The antigen presenting
MHLW	Ministry of Health, Labour and Welfare
CRAds	Cancer-Selective Replicating Adenoviruses
5-FU	5-fluorouracil
CAR	Coxsackie adenovirus receptor
mE1A	Mutant E1A
CPT-11	Camptothecin analogue irinotecan
RGD	Arginine-glycine aspartic acid
pHGG	Pediatric high-grade gliomas
DIPGs	Diffuse intrinsic pontine gliomas
vp	Viral particles
RT	Radiotherapy
4-1BBL	4-1BB ligand
DNX-2440	Delta-24-RGDOX
OX40L	OX40 ligand
pRb	The retinoblastoma tumor suppressor protein
PVSRIPO	Recombinant nonpathogenic polio-rhinovirus chimera
CD	Cytosine deaminase
Toca FC	Prodrug 5-fluorocytosine
SOC	Standard of care
ZIKV	Zika virus
GSCs	Glioblastoma multiforme stem cells
3’UTR	3’ untranslated region
BBB	Blood-brain barrier
TME	Tumor microenvironment
CED	Convection-enhanced delivery
