# Corrigendum: Advances in oncolytic herpes simplex virus and adenovirus therapy for recurrent glioma

**DOI:** 10.3389/fimmu.2023.1352909

**Published:** 2023-12-19

**Authors:** Mingming Hu, XuLiang Liao, Yi Tao, Yaohui Chen

**Affiliations:** ^1^Institute of Thoracic Oncology, West China Hospital, Sichuan University, Chengdu, China; ^2^Frontiers Science Center for Disease-Related Molecular Network, West China Hospital, Sichuan University, Chengdu, China; ^3^Department of Thoracic Surgery, West China Hospital, Sichuan University, Chengdu, China

**Keywords:** oncolytic viruses, cancer therapy, recurrent gliomas, oncolytic herpes simplex virus, adenovirus therapy

In the published article, there was an error in the **Funding** statement. We overlooked the fund that supported this article.

The correct Funding statement appears below:

“This work was supported by the 1.3.5 project for disciplines of excellence, West China Hospital, Sichuan University (Y.C., ZYJC21015).”

The authors apologize for this error and state that this does not change the scientific conclusions of the article in any way. The original article has been updated.

